# A Dentigerous Cyst Associated With a Developing Mandibular Premolar in a Pediatric Patient: A Case Report of a Rare Entity

**DOI:** 10.7759/cureus.107134

**Published:** 2026-04-16

**Authors:** Debadrita Ghosh, Uday Mukherjee, Chitrita Gupta Mukherjee

**Affiliations:** 1 Department of Pedodontics and Preventive Dentistry, Family Dental Clinic, Kolkata, IND; 2 Department of Dentistry, KPC Medical College and Hospital, Kolkata, IND; 3 Department of Pedodontics and Preventive Dentistry, Buddha Institute of Dental Sciences and Hospital, Patna, IND

**Keywords:** dentigerous cyst, enucleation, mixed dentition, odontogenic cyst, pediatric dentistry

## Abstract

Dentigerous cysts are developmental odontogenic cysts commonly associated with impacted teeth; however, their occurrence in young children involving developing premolars is relatively rare. This report describes the case of an eight-year-old male who presented with pain and swelling in the lower left posterior region of the jaw. Clinical examination revealed a grossly carious primary molar with buccal cortical expansion. Radiographic findings showed a well-defined unilocular radiolucency associated with the crown of the developing mandibular second premolar, causing displacement of the tooth bud. Based on the clinical and radiographic features, a dentigerous cyst of inflammatory origin was diagnosed. The lesion was managed by surgical enucleation, extraction of the affected primary tooth, and removal of the displaced permanent tooth germ. Histopathological examination confirmed the diagnosis. Postoperative healing was uneventful. This report highlights the importance of early diagnosis and timely management of odontogenic cysts in children to prevent complications.

## Introduction

Dentigerous cysts are the second most common type of odontogenic cysts, accounting for approximately 20-24% of all jaw cysts, followed by radicular cysts [1,2]. They are developmental in origin and arise due to the accumulation of fluid between the reduced enamel epithelium and the crown of an unerupted or impacted tooth. These cysts are most frequently associated with impacted mandibular third molars, maxillary canines, and mandibular premolars, typically occurring in the second and third decades of life [3]. However, their occurrence in the pediatric population, particularly involving developing premolars, is relatively uncommon and is often associated with inflammatory processes arising from non-vital primary teeth [4].

In children, dentigerous cysts may present as either developmental or inflammatory variants, with the latter being more prevalent owing to periapical infection from carious or non-vital deciduous teeth. Clinically, these cysts are often asymptomatic and are usually discovered incidentally during routine radiographic examinations [4,5]. However, larger lesions may present with cortical expansion, facial asymmetry, delayed eruption of permanent teeth, or displacement of adjacent structures. Radiographically, a dentigerous cyst typically appears as a well-defined unilocular radiolucency associated with the crown of an unerupted tooth, attached at the cementoenamel junction [6].

Early diagnosis and appropriate management are essential to prevent complications such as jaw expansion, pathological fractures, and potential transformation into more aggressive lesions [7]. Treatment modalities range from conservative approaches such as marsupialization to more definitive surgical enucleation, depending on the size, location, and involvement of adjacent structures [2]. This case report aims to present a rare occurrence of a dentigerous cyst associated with a developing mandibular premolar in a young child, highlighting its clinical, radiographic, and surgical management, along with a review of the relevant literature.

## Case presentation

An eight-year-old male patient presented to the Department of Pedodontics and Preventive Dentistry, Buddha Institute of Dental Sciences and Hospital, Patna, India, with a chief complaint of pain and swelling in the lower left posterior region of the jaw for the past two to three weeks. The pain was described as intermittent and dull in nature and was exacerbated during mastication. There was no associated history of trauma, fever, or systemic illness, and the patient’s medical and family history were non-contributory.

**Figure 1:**
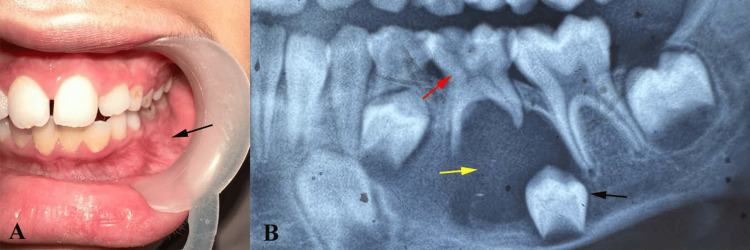
Intraoral exam and radiographic evaluation (A) Intraoral swelling in the posterior mandible obliterating buccal sulcus (black arrow) at the deciduous second molar. (B) Orthopantomogram showed a radiolucent cystic area of 2 x 3 cm in size (yellow arrow), a displaced tooth bud of the second premolar (black arrow), and an infected carious deciduous second molar (red arrow) Original images of the patient; used with the patient's permission

Extraoral examination revealed mild facial asymmetry on the left side, characterized by diffuse swelling over the lower third of the face. The overlying skin appeared normal, with no signs of erythema, sinus formation, or discharge. Palpation revealed a firm, non-fluctuant swelling with mild tenderness. No regional lymphadenopathy was detected. Intraoral examination revealed that the patient was in the mixed-dentition stage. A grossly carious mandibular left second primary molar (tooth 75) was present. The teeth were nonvital on examination. There was noticeable buccal cortical expansion from the mandibular left second primary molar to the mandibular left permanent first molar region, with obliteration of the buccal vestibule. The overlying mucosa appeared intact, without ulceration or sinus tract formation (Figure 1A). On palpation, the area was slightly tender, and the cortical expansion suggested an underlying cystic pathology.

Radiographic evaluation using an orthopantomogram (OPG) and an intraoral periapical radiograph (IOPA) revealed a well-defined (2 × 3 cm) unilocular radiolucent lesion in the left mandibular posterior region (Figure 1B). The lesion was associated with the crown of the unerupted, developing mandibular second premolar (tooth 35), enclosing it and appearing to attach to the cemento-enamel junction. The tooth bud was displaced inferiorly, along with thinning of the surrounding cortical bone. The radiographic features were characteristic of a dentigerous cyst. However, the involvement of a developing premolar in a young child and the relatively large size of the lesion made this presentation atypical. Based on the clinical and radiographic findings, a provisional diagnosis of a dentigerous cyst associated with tooth 35 was made. Differential diagnosis included a radicular cyst arising from the infected primary molar (tooth 75), an odontogenic keratocyst, and a unicystic ameloblastoma.

**Figure 2:**
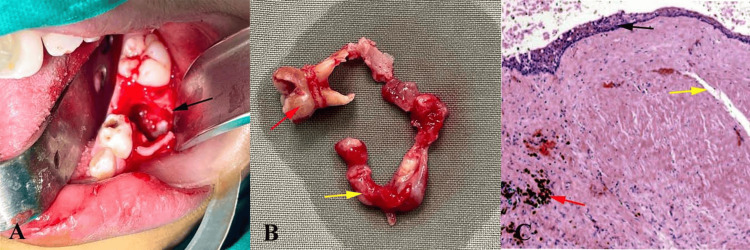
Intraoperative and hispathological images (A) Surgical enucleation of cystic lesion (black arrow). (B) Excised cystic soft tissue specimen (yellow arrow) attached with deciduous molar tooth (red arrow). (C) Hematoxylin and eosin-stained section at 10x magnification showed a thin stratified squamous epithelial lining (black arrow) supported by connective tissue (yellow arrow) with inflammatory infiltrate (red arrow) Original images of the patient; used with the patient's permission

Conservative surgical management was planned under appropriate local anesthesia, considering the age of the patient and the extent of the lesion. Enucleation was preferred over marsupialization because of the well-encapsulated nature of the lesion, the need for definitive management, and concerns regarding patient compliance with prolonged decompression therapy. Enucleation of the cystic lesion was performed along with the extraction of the associated primary tooth (tooth 75) and the removal of the involved permanent tooth bud (tooth 35) because of its marked displacement, unfavorable eruption path, and extensive involvement within the cystic lesion, which rendered functional preservation unlikely. A well-encapsulated cystic lining was identified and completely excised intraoperatively. The surgical site was irrigated and closed (Figure 2A).

The excised specimen consisted of a cystic sac attached to the crown of the developing tooth (Figure 2B) and was sent for histopathological examination. Microscopic evaluation revealed a thin, non-keratinized, stratified squamous epithelial lining with a connective tissue stroma, consistent with the diagnosis of a dentigerous cyst (Figure 2C). Postoperative healing was uneventful, and the patient was followed up regularly for six months. Clinical and radiographic evaluations during subsequent visits demonstrated satisfactory healing, with no evidence of recurrence.

## Discussion

Dentigerous cysts are the most common developmental odontogenic cysts. They most frequently involve the mandibular third molars and maxillary canines in adults, and their occurrence in association with developing premolars in young pediatric patients is notably uncommon and clinically significant. The present case describes a large dentigerous cyst in an eight-year-old male, associated with the developing mandibular second premolar, arising in the context of a deeply carious and non-vital mandibular second primary molar (75). This presentation is consistent with the well-established, though underappreciated, pathogenic pathway in which periapical infection originating from a primary molar propagates toward the subjacent developing permanent tooth follicle, culminating in cystic transformation - a process sometimes termed an "inflammatory dentigerous cyst" or "paradental cyst" depending on the histopathological findings [8,9].

The association between infected primary molars and cyst formation around successor premolars has been increasingly documented in pediatric dental literature. Periapical inflammatory exudate from a carious primary tooth can infiltrate the dental follicle of the permanent successor, inciting epithelial proliferation within the follicular remnants and ultimately generating cystic enlargement that radiographically mimics a classic developmental dentigerous cyst [9,10]. Several case series have reported this inflammatory etiology specifically in children below the age of ten years, underscoring that a large pericoronal radiolucency in a child should not be reflexively attributed to a developmental origin without careful clinical and etiological correlation [10,11]. In the present case, the gross carious destruction and non-vitality of tooth 75, combined with the radiographic features of the lesion attached to the cementoenamel junction of the unerupted premolar, strongly support an inflammatory origin, which is directly related to both the biological behavior of the lesion and the prognostic expectations following surgical management.

Radiographically, dentigerous cysts typically present as well-defined, corticated unilocular radiolucencies enclosing the crown of an unerupted tooth with attachment at the cementoenamel junction, features that were classically demonstrated in this case [12,13]. However, the differential diagnosis in such presentations must systematically include the odontogenic keratocyst, which may exhibit histologically identical radiographic appearances but carries significantly higher recurrence rates and distinct biological behavior, mandating a different management philosophy [14]. Unicystic ameloblastoma, although rare in children, must equally be excluded given its capacity to mimic a dentigerous cyst both radiographically and clinically, yet it carries substantially greater morbidity if inadequately treated [15]. In the present case, the definitive diagnosis was based on histopathological confirmation, which revealed a thin non-keratinized stratified squamous epithelial lining without atypia, keratinization, or mural ameloblastomatous proliferation, features unequivocally consistent with a simple dentigerous cyst.

Management of dentigerous cysts in growing patients requires a careful balance between achieving definitive eradication of the lesion and preserving the developmental and functional integrity of the dentition. Marsupialization has been advocated as a conservative approach in young patients, as it decompresses the cyst, permits the associated tooth to potentially re-erupt, and reduces the extent of surgical trauma to adjacent developing structures [11,16]. However, marsupialization demands a high degree of patient cooperation for prolonged postoperative maintenance and cavity irrigation, which is frequently challenging in young pediatric patients.

Furthermore, in cases where the involved permanent tooth exhibits marked displacement, unfavorable angulation, and extensive cystic envelopment, the likelihood of spontaneous re-eruption following marsupialization is markedly diminished [13,16]. In the present case, enucleation was deemed the most appropriate definitive treatment modality. The decision to remove the developing premolar tooth bud alongside the cystic lining and the offending primary molar was guided by its significantly inferior displacement, attendant risk of damage to adjacent anatomical structures, and low probability of functional eruption. This approach is consistent with published management guidelines advocating tooth bud removal when the displacement is severe, and the eruption potential is negligible [2,14].

From a developmental standpoint, the premature loss of the permanent second premolar in young patients has significant orthodontic implications, including space loss, mesial drift of the permanent first molar, and potential midline deviation. Timely orthodontic consultation and space management planning are therefore essential components of the long-term treatment strategy following surgical intervention [17,11]. Early diagnosis, facilitated by routine radiographic screening in children presenting with chronic dental infections, is paramount for preventing the extensive destruction observed in this case. Several authors have emphasized that the delayed diagnosis of inflammatory follicular cysts in children leads to progressive bone destruction, displacement of multiple teeth, and increased complexity of surgical management [8,9,2]. This case reinforces the critical importance of prompt and thorough radiographic evaluation of all chronically infected primary teeth in pediatric patients, recognizing that periapical pathology extending to the permanent successor represents a dental emergency warranting immediate intervention.

## Conclusions

This report demonstrates the rare occurrence of an inflammatory dentigerous cyst associated with a developing mandibular second premolar in an eight-year-old child, secondary to a grossly carious primary molar. The lesion exhibited significant cortical expansion and displacement of the tooth bud, necessitating surgical enucleation along with removal of the involved permanent tooth germ owing to its poor eruptive prognosis. Early diagnosis through radiographic evaluation of infected primary teeth plays a key role in preventing further complications. The report emphasizes the need for careful monitoring of carious or treated deciduous teeth and timely intervention to ensure favorable healing and prevent adverse effects on the developing permanent dentition.
